# Bridge to Better Care: Investigating Transient Ischemic Attack (TIA) Management Expertise Among Primary Healthcare Providers in Al-Ahsa, Saudi Arabia

**DOI:** 10.7759/cureus.50420

**Published:** 2023-12-12

**Authors:** Sadiq Al Salman, Abdulmuhsin A Al Sultan, Mohammed A Aldawood, Hawra K Alradhi, Maryam A AlMuhaish, Salma A Alsumaeel

**Affiliations:** 1 Neurology, King Fahad General Hospital, Al-Ahsa, SAU; 2 College of Medicine, King Faisal University, Al-Ahsa, SAU; 3 Neurology, King Fahad Specialist Hospital, Dammam, SAU

**Keywords:** saudi arabia, nurses, doctors, management, transient, ischemic attack

## Abstract

Introduction

Transient ischemic attacks (TIAs) are brief episodes of neurological impairment caused by reduced blood flow to the brain, spinal cord, or retina, typically lasting under an hour. Recent advances in neuroimaging suggest that some TIAs may actually be small strokes with resolved symptoms. This study focuses on assessing the knowledge and management of TIAs among primary care physicians and nurses in Al-Ahsa, Saudi Arabia.

Methodology

This is a cross-sectional study, conducted in Al-Ahsa, Saudi Arabia, during the period July to August 2023. Data were collected using an electronic questionnaire and was analyzed using IBM SPSS Statistics for Windows, version 27.0.1 (released 2020, IBM Corp., Armonk, New York, United States).

Results

Among the participants, 64.0% correctly identified TIA as an ischemic neurological deficit. However, only 20.2% provided correct responses for all TIA symptoms. Regarding diagnostic tests, 47.4% acknowledged the need for neuroimaging immediately after TIA, while 17.5% recognized the importance of ultrasonography of the supra-aortic trunks. In terms of TIA management, 38.6% preferred referral to the emergency service, and 41.2% correctly perceived the risk of TIA recurrence as similar to that of established cerebral ischemic stroke. Significant disparities were observed in the recognition of TIA symptoms, with physicians outperforming nurses, particularly in identifying motor deficits (82.4% vs. 65.2%) and speech alterations (86.8% vs. 76.1%, p = 0.004). However, nurses exhibited better knowledge in recognizing the need for a neuroimaging test (48.5% vs. 45.7%, p = 0.849) and the urgency of conducting a transcranial Doppler (TCD) (19.1% vs. 23.9%, p = 0.641).

Conclusion

A considerable proportion of healthcare providers demonstrate a good understanding of TIA definition and management. However, the lack of significant predictors for good knowledge and attitude suggests the need for more comprehensive strategies to enhance TIA management expertise across healthcare professionals.

## Introduction

A transient ischemic attack (TIA) is an episode of temporary neurologic impairment that comes from focal ischemia of the brain, spinal cord, or retina but does not include acute tissue infarction. It usually lasts less than an hour [1]. Advances in neuroimaging imply that many of these cases may represent small strokes with resolved symptoms rather than true TIAs, although the traditional definition of TIA includes symptoms lasting as long as 24 hours [2].

Both TIA and small ischemic stroke are linked to brain malfunction in a specific location brought on by a localized decrease in blood flow and manifested as either temporary or insignificant clinical symptoms [3].

The available estimated yearly incidence rates of TIA in Western countries range from 29.0 to 61.0 cases per 100,000 [4,5]. With risk estimates within three months ranging from 7.5% to 17.3%, TIA is a recognized predictor of future ischemic stroke [6,7]. The better identification and management of vascular risk factors may have modified TIA epidemiology in recent years [8].

The American Society of Anesthesiologists (ASA)'s most recent recommendations classify risk variables using an evidence-based methodology and with some flexibility: age, sex, race, and significant family history are non-modifiable; tobacco use, obesity, physical inactivity, cardiovascular disease, atrial fibrillation, diabetes mellitus, arterial hypertension, and peripheral arterial disease are well documented and modifiable; and migraine history, obstructive sleep apnea, sleep patterns, and high-risk alcohol consumption are potentially modifiable [9,10].

Given the fact that TIA signs and symptoms are transient, less accurate than those in patients with stroke who present with well-established clinical presentation, the diagnosis of TIA might be challenging [11].

The signs and symptoms of TIA are manifested based on the vascular supply of the affected area. Eighty percent of TIAs and strokes occur in the anterior circulation, which carries about 80% of cerebral blood flow [12]. The anterior circulation of the brain is a branch of the internal carotid artery. Anterior circulation branches off further to give anterior and middle cerebral arteries and their tributaries. The remaining 20% of brain areas are supplied by posterior circulation, which is formed by the two vertebral arteries that combine to give rise to the basilar artery. Thus, 20% of TIAs affect these vertebrobasilar territories [13]. 

The clinical manifestations of TIA include hemiparesis and other types of motor dysfunction, being the most common presentation; somatosensory symptoms; dysarthria and/or aphasia; monocular blindness, i.e, amaurosis fugax resulting from an occlusion in of the ophthalmic arteries, which receive their blood supply directly from internal carotid artery; hemivisual field defects; dizziness; diplopia accompanied or preceded by posterior circulation-suggestive symptoms, such as vertigo, dysarthria or ataxia, headache, limb shaking, and other involuntary movements; cognitive and emotional symptoms; and loss of consciousness or syncope [11].

Up to 20% of TIA patients proceed to ischemic stroke. Thus, a proper identification of TIA signs and symptoms and rapid assessment by primary care physicians are crucial [14]. In this study, we aim to assess the knowledge and management of TIAs among primary care physicians and nurses in Al-Ahsa, Saudi Arabia, through a survey.

## Materials and methods

The study was approved by the Research Ethics Committee at King Fahad Hospital Hofuf, Saudi Arabia, on November 2023 (approval no. 58B-EP-2023). 

Statistical analysis

Both descriptive and inferential statistical analyses of the data were carried out. Simple descriptive statistics of the sociodemographic characteristics and other categorical variables in the form of frequencies and percentages were calculated and tabulated. For continuous variables, means and standard deviations were reported as measures of central tendency and dispersion, respectively.

For the seven questions assessing knowledge of TIA, a score of 1 was assigned for each correct response, and the total score of each participant was calculated. A score of 4 or greater was considered as good knowledge. Furthermore, referral to the emergency department to carry out an urgent CT scan was considered good attitude.

Both univariate and multivariate inferential statistical analysis was performed. Univariate analysis involved using Fischer's exact test for categorical variables and independent samples t-test for continuous variables. Multivariate analysis was done through binary logistic regression to identify predictors of good knowledge and attitude toward TIA patients.

Significance was established at a p-value of 0.05 or less, indicating a 95% confidence interval (CI). All statistical calculations were performed using IBM SPSS Statistics for Windows, version 27.0.1 (released 2020; IBM Corp., Armonk, New York, United States).

## Results

Sociodemographic characteristics

Table 1 presents the sociodemographic characteristics of the 114 study participants, comprising 68 doctors and 46 nurses. Doctors were, on average, younger (N =68) (mean age = 31.8 years) compared to nurses (N = 46) (mean age = 35.4 years), with a statistically significant age difference (P = 0.029). More nurses (67.4%) (N = 31) were female, while more doctors (60.3%) (N = 41) were male, showing a significant gender difference (P = 0.004).

**Table 1:** Sociodemographic characteristics of the participants *P < 0.05, significant; F: Fischer's exact test, t: independent samples t-test

		Doctor (N = 68)		Nurse (N = 46)		Total (N = 114)		P value^F/t^
		Count	%	Count	%	Count	%	
Age years - Mean (SD)		31.8 (7.1)		35.4 (9.3)		33.2 (8.2)		0.029*
Age category	20-29 years	32	47.1%	14	30.4%	46	40.4%	0.067
	30-39 years	26	38.2%	18	39.1%	44	38.6%	
	40-49 years	9	13.2%	9	19.6%	18	15.8%	
	>50 years	1	1.5%	5	10.9%	6	5.3%	
Gender	Female	27	39.7%	31	67.4%	58	50.9%	0.004*
	Male	41	60.3%	15	32.6%	56	49.1%	
Years of practice	5-10 years	16	23.5%	10	21.7%	26	22.8%	0.208
	Less than 5 years	37	54.4%	19	41.3%	56	49.1%	
	More than 5 years	15	22.1%	17	37.0%	32	28.1%	
Total		68	59.6%	46	40.4%	114	100.0%	

Age categories showed no significant difference (P = 0.067), with the majority in the 20-29 (47.1%, N = 32 doctors; 30.4%, N = 14 nurses) and 30-39 (38.2%, N = 26 doctors; 39.1%, N = 18 nurses) age groups. Years of practice indicated no significant variation (P = 0.208), with 49.1% (N = 56) having less than five years of experience and 28.1% (N = 32) having more than five years, although a higher proportion of doctors (54.4%) (N = 37) had less than five years of practice.

In summary, this table highlights that doctors and nurses differed significantly in age and gender but not in years of practice.

Survey responses

Regarding the definition of TIAs, 64.0% (N = 73) correctly identified it as an ischemic neurological deficit lasting less than one hour or a transient deficit lasting less than 24 hours. While the participants recognized various TIA symptoms, only 20.2% (N = 23) provided correct responses for all.

Concerning diagnostic tests, 47.4% (N = 54) acknowledged the need for neuroimaging immediately after TIA, and 17.5% (N = 20) recognized the importance of ultrasonography of the supra-aortic trunks. For the transcranial doppler (TCD), 21.1% (N = 24) emphasized its prompt implementation.

Regarding TIA management, 38.6% (N = 44) preferred referral to the emergency service, while 41.2% (N = 47) correctly perceived the risk of TIA recurrence as similar to that of established cerebral ischemic stroke. In total, 22.8% (N = 26) demonstrated good knowledge of TIA management, and 21.1% (N = 24) exhibited a positive attitude toward referring patients for a CT scan in an emergency (Table 2).

**Table 2:** Survey responses and differences between doctors and nurses *P < 0.05, significant; F: Fischer's exact test

		Doctors		Nurses		Total		P value^F^
		Count	Column %	Count	Column %	Count	Column %	
Definition of TIA	Any transient neurological deficit regardless of the length of symptoms	13	19.1%	11	23.9%	24	21.1%	
	Any transient neurological deficit which experiences a significant improvement	8	11.8%	9	19.6%	17	14.9%	
	Ischemic neurological deficit of less than one hour duration*	13	19.1%	8	17.4%	21	18.4%	
	Transient ischemic neurological deficit of less than 24 hours duration*	34	50.0%	18	39.1%	52	45.6%	
	Correct response	47	69.1%	26	56.5%	73	64.0%	0.233
Symptoms of TIA	Motor deficit*	56	82.4%	30	65.2%	86	75.4%	
	Sensory deficit*	52	76.5%	26	56.5%	78	68.4%	
	Speech alteration*	59	86.8%	35	76.1%	94	82.5%	
	Loss of consciousness*	38	55.9%	21	45.7%	59	51.8%	
	Visual field deficit or amaurosis*	52	76.5%	17	37.0%	69	60.5%	
	Isolated vertigo*	33	48.5%	11	23.9%	44	38.6%	
	Correct Response	20	29.4%	3	6.5%	23	20.2%	0.004*
Neuroimaging	A neuroimaging test must be conducted as soon as possible after TIA*	33	48.5%	21	45.7%	54	47.4%	
	Conducting a neuroimaging test is necessary but not urgent	14	20.6%	14	30.4%	28	24.6%	
	It is not essential for the diagnosis and treatment of these patients to perform a cranial CT or brain MRI	21	30.9%	11	23.9%	32	28.1%	
	Correct response	33	48.5%	21	45.7%	54	47.4%	0.849
Ultrasonography of the supra-aortic trunks (SAT duplex scan)	An SAT duplex must be conducted as soon as possible after TIA*	15	22.1%	5	10.9%	20	17.5%	
	Conducting an ultrasound test is necessary but not urgent	33	48.5%	21	45.7%	54	47.4%	
	It is essential for the diagnosis and treatment of these patients to perform a SAT duplex scan	20	29.4%	20	43.5%	40	35.1%	
	Correct Response	15	22.1%	5	10.9%	20	17.5%	0.140
Transcranial doppler	A TCD must be conducted as soon as possible after TIA*	13	19.1%	11	23.9%	24	21.1%	
	Conducting a TCD is necessary but not urgent	19	27.9%	16	34.8%	35	30.7%	
	I do not know this diagnostic test	36	52.9%	19	41.3%	55	48.2%	
	Correct response	13	19.1%	11	23.9%	24	21.1%	0.641
Management of patients with TIA	Ordinary derivation to outpatient neurology consultation	3	4.4%	2	4.3%	5	4.4%	
	Preferential referral to outpatient neurology consultation	12	17.6%	15	32.6%	27	23.7%	
	Referral to the emergency service*	31	45.6%	13	28.3%	44	38.6%	
	Start of antiaggregation and request for complementary tests by the primary consultation	22	32.4%	16	34.8%	38	33.3%	
	Correct response	31	45.6%	13	28.3%	44	38.6%	0.078*
Recurrence after TIA	Is higher than that of a patient with established cerebral ischemic stroke*	29	42.6%	18	39.1%	47	41.2%	
	Is lower than that of a patient with established cerebral ischemic stroke	10	14.7%	10	21.7%	20	17.5%	
	Is the same as that of a patient with established cerebral ischemic stroke	29	42.6%	18	39.1%	47	41.2%	
	Correct response	29	42.6%	18	39.1%	47	41.2%	0.846
Good knowledge of TIA management (≥4 correct responses)		18	26.5%	8	17.4%	26	22.8%	0.363
Good attitude (referral to emergency service to carry out a CT scan)		17	25.0%	7	15.2%	24	21.1%	0.247

Difference between doctors and nurses

Table 2 also presents survey responses, highlighting differences between doctors (N = 68) (59%.6) and nurses (N = 46) (40.3%) in terms of their understanding of TIA management, along with associated p values for significance. Regarding the definition of TIA, 69.1% (N = 47) of doctors correctly identified it as an ischemic neurological deficit of less than one/24-hour duration, whereas 56.5% (N = 26) of nurses selected the correct option (p = 0.233).

Significant differences were observed in their recognition of TIA symptoms. Doctors outperformed nurses, particularly in identifying motor deficits (82.4% vs. 65.2%; N = 56 vs. N = 30) and speech alterations (86.8% vs. 76.1%, N = 59 vs. N = 35; p = 0.004). However, nurses exhibited better knowledge in recognizing the need for a neuroimaging test (45.7% vs. 48.5%, N = 54 vs. N = 33; p = 0.849) and the urgency of conducting a TCD (23.9% vs. 19.1%, N = 11 vs. N = 13; p = 0.641) although not statistically significant.

In terms of management, doctors were more inclined to refer patients to the emergency service (45.6% vs. 28.3%, N = 31 vs. N = 13; p = 0.078), while nurses incorrectly preferred outpatient neurology consultation (32.6%) (N = 15). Both groups showed similar attitudes toward TIA recurrence, with no significant differences observed (p = 0.846). Notably, 26.5% (N = 18) of doctors and 17.4% (N = 8) of nurses demonstrated good knowledge of TIA management (p = 0.363), but no significant difference was observed. Similarly, 25% (N = 17) of doctors and 15.2% (N = 24) of nurses exhibited a positive attitude toward referring patients to the emergency service for a CT scan (p = 0.247).

Variables associated with correct knowledge of TIA among doctors

Table 3 present the variables associated with good TIA knowledge among doctors, with a focus on different aspects of their knowledge and demographics.

**Table 3:** Variables associated with good TIA knowledge among physicians a. Position/Title  = Physician; F: Fischer's exact test, t: independent samples t-test; TIA: transient ischemic attack

		Good knowledge of TIA management				P value^F/t^	Definition of TIA				P value^F/t^	Symptoms of TIA				P value^F/t^	Neuroimaging				P value^F/t^
		<4 Correct N		>=4 Correct N			Incorrect N		Correct N			Incorrect N		Correct N			Incorrect N		Correct N		
		N	%	N	%		N	%	N	%		N	%	N	%		N	%	N	%	
Age years - Mean (SD)		32.2 (7.7)		30.7 (5.2)		0.380	34.8 (7.6)		30.4 (6.6)		0.029*	32.8 (7.6)		29.3 (5.3)		0.033*	31.9 (7.7)		31.7 (6.6)		0.941
Age category	20-29 years	24	48%	8	44.4%	0.570	6	28.6%	26	55.3%	0.030*	21	43.8%	11	55%	0.604	17	48.6%	15	45.5%	0.972
	30-39 years	17	34%	9	50%		9	42.9%	17	36.2%		18	37.5%	8	40%		13	37.1%	13	39.4%	
	40-49 years	8	16%	1	5.6%		6	28.6%	3	6.4%		8	16.7%	1	5%		4	11.4%	5	15.2%	
	>50 years	1	2%	0	0%		0	0%	1	2.1%		1	2.1%	0	0%		1	2.9%	0	0%	
Gender	Female	21	42%	6	33.3%	0.584	5	23.8%	22	46.8%	0.108	20	41.7%	7	35%	0.786	14	40%	13	39.4%	1.000
	Male	29	58%	12	66.7%		16	76.2%	25	53.2%		28	58.3%	13	65%		21	60%	20	60.6%	
Years of practice	Less than 5 years	27	54%	10	55.6%	0.659	8	38.1%	29	61.7%	0.083	23	47.9%	14	70%	0.209	19	54.3%	18	54.5%	0.492
	5-10 years	13	26%	3	16.7%		5	23.8%	11	23.4%		12	25%	4	20%		10	28.6%	6	18.2%	
	More than 10 years	10	20%	5	27.8%		8	38.1%	7	14.9%		13	27.1%	2	10%		6	17.1%	9	27.3%	

In Table 3, age was not significantly associated with good TIA knowledge (p = 0.380), as indicated by the mean age of 32.2 years for those with less than four correct responses and 30.7 years for those with greater than or equal to four correct responses. However, a significant difference was observed in the definition of TIA knowledge (p = 0.029*), with those aged 30.4 years among those with good knowledge, compared to 34.8 years among those without. In addition, the participants in the 20-29-year age category showed a significantly higher likelihood of having a good understanding of TIA symptoms (55.3%) (N = 26) compared to the other age groups (p = 0.030*).

Regarding gender, there were no significant differences in TIA knowledge between male and female doctors. In terms of years of practice, no significant associations were found between the duration of practice and TIA knowledge in the variables assessed.

Table 4 examines knowledge associations related to ultrasound studies of the supra-aortic trunks, TCD, management of TIA patients, and recurrence of TIA among doctors. Similar to Table 5, age did not significantly affect knowledge in these categories. Gender and years of practice also did not show significant associations with TIA knowledge in these areas. 

**Table 4:** Variables associated with good TIA knowledge among physicians a. Position / Title  = Physician; F: Fischer's exact test; t: independent samples t-test; TIA: transient ischemic attack

		Ultrasound study of the supra-aortic trunks				P value^F/t^	Transcranial doppler				P value^F/t^	Management of patients with TIA				P value^F/t^	Recurrence after TIA				P value^F/t^
		Incorrect N		Correct N			Incorrect N		Correct N			Incorrect N		Correct N			Incorrect N		Correct N		
		N	%	N	%		N	%	N	%		N	%	N	%		N	%	N	%	
Age years - Mean (SD)		31.9 (6.7)		31.4 (8.7)		0.838	31.4 (7.0)		33.4 (7.8)		0.418	31.6 (8.1)		32.0 (5.8)		0.852	31.9 (7.7)		31.6 (6.4)		0.833
Age category	20-29 years	24	45.3%	8	53.3%	0.278	27	49.1%	5	38.5%	0.750	20	54.1%	12	38.7%	0.339	17	43.6%	15	51.7%	0.881
	30-39 years	22	41.5%	4	26.7%		20	36.4%	6	46.2%		11	29.7%	15	48.4%		15	38.5%	11	37.9%	
	40-49 years	7	13.2%	2	13.3%		7	12.7%	2	15.4%		5	13.5%	4	12.9%		6	15.4%	3	10.3%	
	>50 years	0	0%	1	6.7%		1	1.8%	0	0%		1	2.7%	0	0%		1	2.6%	0	0%	
Gender	Female	22	41.5%	5	33.3%	0.766	22	40%	5	38.5%	1.000	15	40.5%	12	38.7%	1.000	14	35.9%	13	44.8%	0.617
	Male	31	58.5%	10	66.7%		33	60%	8	61.5%		22	59.5%	19	61.3%		25	64.1%	16	55.2%	
Years of practice	Less than 5 years	29	54.7%	8	53.3%	0.856	33	60%	4	30.8%	0.123	21	56.8%	16	51.6%	0.805	20	51.3%	17	58.6%	0.222
	5-10 years	13	24.5%	3	20%		12	21.8%	4	30.8%		9	24.3%	7	22.6%		12	30.8%	4	13.8%	
	More than 10 years	11	20.8%	4	26.7%		10	18.2%	5	38.5%		7	18.9%	8	25.8%		7	17.9%	8	27.6%	

Variables associated with correct knowledge of TIA among nurses

Table 5 and Table 6 present variables associated with good TIA knowledge among nurses, focusing on TIA management, definition, symptoms, neuroimaging, ultrasound studies of the supra-aortic trunks, TCD, management of patients with TIA, and recurrence after TIA.

**Table 5:** Variables associated with good TIA knowledge among nurses a. Position/Title  = Nurse; F: Fischer's exact test; t: independent samples t-test

		Good knowledge of TIA management				P value^F/t^	Definition of TIA				P value^F/t^	Symptoms of TIA				P value^F/t^	Neuroimaging				P value^F/t^
		<4 Correct N		>=4 Correct N			Incorrect N		Correct N			Incorrect N		Correct N			Incorrect N		Correct N		
		N	%	N	%		N	%	N	%		N	%	N	%		N	%	N	%	
Age years - Mean (SD)		35.6 (9.7)		34.5 (7.7)		0.737	35.3 (10.4)		35.5 (8.5)		0.931	35.1 (9.5)		39.7 (3.5)		0.132	38.4 (10.1)		31.9 (7.0)		0.014*
Age category	20-29 years	11	28.9%	3	37.5%	0.850	6	30%	8	30.8%	1.000	14	32.6%	0	0%	0.216	6	24%	8	38.1%	0.102
	30-39 years	15	39.5%	3	37.5%		8	40%	10	38.5%		17	39.5%	1	33.3%		8	32%	10	47.6%	
	40-49 years	7	18.4%	2	25%		4	20%	5	19.2%		7	16.3%	2	66.7%		6	24%	3	14.3%	
	>50 years	5	13.2%	0	0%		2	10%	3	11.5%		5	11.6%	0	0%		5	20%	0	0%	
Gender	Female	26	68.4%	5	62.5%	1.000	15	75%	16	61.5%	0.365	28	65.1%	3	100%	0.541	16	64%	15	71.4%	0.591
	Male	12	31.6%	3	37.5%		5	25%	10	38.5%		15	34.9%	0	0%		9	36%	6	28.6%	
Years of practice	Less than 5 years	16	42.1%	3	37.5%	0.694	8	40%	11	42.3%	0.864	19	44.2%	0	0%	0.313	8	32%	11	52.4%	0.353
	5-10 years	9	23.7%	1	12.5%		5	25%	5	19.2%		9	20.9%	1	33.3%		7	28%	3	14.3%	
	More than 10 years	13	34.2%	4	50%		7	35%	10	38.5%		15	34.9%	2	66.7%		10	40%	7	33.3%	

**Table 6:** Variables associated with good TIA knowledge among nurses a. Position/Title  = Nurse; F: Fischer's exact test; t: independent samples t-test; TIA: transient ischemic attack

		Ultrasound study of supra-aortic trunks				P value^F/t^	Transcranial Doppler				P value^F/t^	Management of patients with TIA				P value^F/t^	Recurrence after TIA				P value^F/t^
		Incorrect N		Correct N			Incorrect N		Correct N			Incorrect N		Correct N			Incorrect N		Correct N		
		N	%	N	%		N	%	N	%		N	%	N	%		N	%	N	%	
Age years - Mean (SD)		35.3 (9.6)		36.4 (7.1)		0.759	36.2 (9.9)		32.7 (6.9)		0.203	35.5 (9.7)		35.0 (8.5)		0.852	34.5 (9.6)		36.7 (8.9)		0.436
Age Category	20-29 years	13	31.7%	1	20%	0.909	9	25.7%	5	45.5%	0.483	10	30.3%	4	30.8%	0.965	11	39.3%	3	16.7%	0.200
	30-39 years	15	36.6%	3	60%		14	40%	4	36.4%		12	36.4%	6	46.2%		8	28.6%	10	55.6%	
	40-49 years	8	19.5%	1	20%		7	20%	2	18.2%		7	21.2%	2	15.4%		5	17.9%	4	22.2%	
	>50 years	5	12.2%	0	0%		5	14.3%	0	0%		4	12.1%	1	7.7%		4	14.3%	1	5.6%	
Gender	Female	28	68.3%	3	60%	1.000	23	65.7%	8	72.7%	1.000	22	66.7%	9	69.2%	1.000	20	71.4%	11	61.1%	0.530
	Male	13	31.7%	2	40%		12	34.3%	3	27.3%		11	33.3%	4	30.8%		8	28.6%	7	38.9%	
Years of practice	Less than 5 years	18	43.9%	1	20%	0.519	14	40%	5	45.5%	0.743	14	42.4%	5	38.5%	1.000	14	50%	5	27.8%	0.371
	5-10 years	9	22%	1	20%		7	20%	3	27.3%		7	21.2%	3	23.1%		5	17.9%	5	27.8%	
	More than 10 years	14	34.1%	3	60%		14	40%	3	27.3%		12	36.4%	5	38.5%		9	32.1%	8	44.4%	

In Table 5 and Table 6, the analysis revealed that there were no statistically significant differences in TIA knowledge among nurses based on gender, years of practice, or the majority of age categories. However, a significant difference was observed regarding knowledge of neuroimaging among different ages. Specifically, younger nurses (mean age = 31.97, SD = 7.0) displayed better knowledge of neuroimaging compared to their older counterparts (mean age = 38.4, SD = 10.1; p = 0.014).

Predictors of correct knowledge and management of TIA

Table 7 presents the predictors of good knowledge and attitude regarding TIA among the studied cohort, which includes both doctors and nurses. The table reports the p-values, adjusted odds ratios (AORs), and their corresponding 95% CIs for several factors.

**Table 7:** Predictors of good knowledge and attitude of TIA AOR: adjusted odds ratio; CI: confidence interval; TIA: transient ischemic attack. *P < 0.05, significant

Objective	Cohort studied	Variable	Adjusted odds ratio (AOR)	95% C.I. for AOR		P value
				Lower	Upper	
Good knowledge of TIA (>3 correct answers)	All	Age	0.908	0.815	1.012	0.081
		Males (relative to females)	1.933	0.707	5.290	0.199
		Years of practice (less than 5 years as a reference)				0.142
		5-10 years	1.401	0.301	6.527	0.667
		More than 10 years	4.738	0.863	26.009	0.073
		Physicians (relative to nurses)	1.365	0.491	3.796	0.551
	Physicians	Age	.898	0.782	1.032	0.130
		Males (relative to females)	2.123	0.604	7.459	0.240
		Years of practice (less than 5 years as a reference)				0.309
		5-10 years	1.453	0.225	9.370	0.694
		More than 10 years	4.587	0.555	37.924	0.158
	Nurses	Age	0.920	0.776	1.091	0.339
		Males (relative to females)	1.677	0.299	9.407	0.557
		Years of practice (less than 5 years as a reference)				0.462
		5-10 years	1.401	0.082	23.890	0.816
		More than 10 years	5.135	0.291	90.498	0.264
Good attitude (referral to emergency service to carry out a CT scan)	All	Age	0.973	0.877	1.079	0.605
		Males (relative to females)	1.272	0.465	3.484	0.639
		Years of practice (less than 5 years as a reference)				0.186
		5-10 years	0.322	0.051	2.018	0.226
		More than 10 years	1.580	0.301	8.300	0.589
		Physicians (relative to nurses)	1.777	0.615	5.137	0.288
	Physicians	Age	1.017	0.893	1.159	0.794
		Males (relative to females)	1.288	0.374	4.437	0.689
		Years of practice (less than 5 years as a reference)				0.376
		5-10 years	0.315	0.041	2.427	0.267
		More than 10 years	1.056	0.136	8.209	0.959
	Nurses	Age	0.876	0.704	1.092	0.239
		Males (relative to females)	1.017	0.150	6.921	0.986
		Years of practice (less than 5 years as a reference)				0.653
		5-10 years	.000	.000	.	0.999
		More than 10 years	4.711	0.175	126.634	0.356

For the prediction of good knowledge of TIA, the analysis indicates that age (p = 0.081, AOR = 0.908, 95% CI: 0.815-1.012) is not a significant predictor. Gender, years of practice, and professional background (doctors relative to nurses) also do not appear to be significant factors influencing good knowledge of TIA. However, for individuals with more than 10 years of experience (p = 0.073, AOR = 4.738, 95% CI: 0.863-26.009), there is a trend toward higher odds of having good knowledge compared to those with less than five years of experience, although this trend did not reach statistical significance.

In the context of good attitude toward TIA, the analysis again demonstrates that age (p = 0.605, AOR = 0.973, 95% CI: 0.877-1.079) does not significantly influence attitudes. Gender (p = 0.639, AOR = 1.272, 95% CI: 0.465-3.484) also does not appear to be a significant predictor of good attitude. Similarly, years of practice do not significantly impact attitudes. However, doctors, when compared to nurses (p = 0.288, AOR = 1.777, 95% CI: 0.615-5.137), exhibit a trend toward higher odds of having a good attitude, although this trend is not statistically significant.

When looking at the predictions separately for doctors and nurses, similar trends were observed in both groups, with no significant predictors for good knowledge and attitude among doctors or nurses based on age, gender, or years of practice.

In summary, the analysis suggests that the factors of age, gender, years of practice, and professional background do not significantly predict good knowledge and attitude toward TIA among the studied cohort of doctors and nurses. Further research and larger sample sizes may be necessary to draw more definitive conclusions regarding these predictors.

## Discussion

Our study shed light on the knowledge and practices of healthcare providers regarding TIA management. The study revealed notable sociodemographic differences between doctors and nurses. Doctors were generally younger and more likely to be male, while nurses were slightly older and predominantly female. These differences could influence their approach to TIA management, emphasizing the need for targeted educational interventions. The majority of both doctors and nurses demonstrated a reasonable understanding of the definition of TIA, which suggests that both groups have a solid grasp of the basic definition of TIA [15]. A significant proportion of participants demonstrated a correct understanding of TIA in terms of duration and symptoms. However, there is room for improvement, particularly regarding knowledge of diagnostic tests and appropriate management strategies. In general, doctors and nurses exhibited differences in their understanding of TIA management.

Doctors showed a better understanding of TIA symptoms, especially motor deficits and speech alterations, which indicates that doctors have more comprehensive training in recognizing the clinical manifestations of TIA and also highlighting the need for further education for nurses in this aspect [14]. Nurses exhibited a relatively stronger understanding of the need for neuroimaging tests and the urgency of conducting a TCD, as also evident by a study providing importance of TCD in acute stroke, conducted by Sharma et al. (2008) [16]. This suggests that nurses may be more attuned to the diagnostic aspects of TIA evaluation, possibly due to their roles in patient assessment and care coordination, suggesting potential areas for improving doctors’ awareness in these domains [16]. There is a notable gap in the understanding of the urgency of neuroimaging immediately after a TIA event, emphasizing the necessity for increased awareness and education [17].

Doctors were more inclined to refer TIA patients to emergency services, underscoring their proactive approach to urgent care. However, there is a need to align practices with established guidelines [18]. Both doctors and nurses displayed similar attitudes toward the risk of TIA recurrence, with no significant differences observed. This suggests that both groups perceive the risk of TIA recurrence similarly, which is a positive aspect as it reflects a unified understanding of the condition’s potential severity [19].

Age was not a significant factor in determining TIA knowledge among doctors. However, there was a noteworthy difference in the understanding of TIA symptoms, with younger doctors having a significantly better grasp of TIA symptoms compared to other age groups. This suggests that younger doctors may have received more recent training or education on TIA symptoms [20]. No significant differences in TIA knowledge between male and female doctors were found, indicating that gender does not play a role in TIA-related knowledge among this group of healthcare providers. Interestingly, the duration of practice did not show significant associations with TIA knowledge among doctors. This suggests that regardless of their years of experience, doctors in the study had similar levels of TIA knowledge [21].

Similarly, among nurses, age did not significantly impact TIA knowledge, except for knowledge related to neuroimaging. Younger nurses demonstrated better knowledge of neuroimaging compared to their older counterparts. This may be due to younger nurses having received more up-to-date training in this area [22].

When considering both doctors and nurses together, the study found that age, gender, and years of practice did not significantly predict good knowledge or a positive attitude toward TIA. This implies that regardless of their age, gender, or years of experience, there is a relatively consistent baseline level of TIA knowledge and attitudes among the studied healthcare professionals. This finding suggests that factors beyond demographic characteristics play a more significant role in shaping their knowledge and attitudes [23]. However, individuals with more than 10 years of experience showed a trend toward higher odds of having good TIA knowledge.

Similarly, doctors exhibited a trend toward a more positive attitude compared to nurses. It suggests that a longer duration of practice may provide healthcare providers with additional exposure to TIA cases and clinical scenarios, leading to a deeper understanding of the condition over time [24]. This could indicate that experience is an important factor in acquiring expertise in TIA management [25].

The study has several limitations that should be acknowledged. The sample size of 114 participants, while informative, may not fully represent the entire healthcare workforce in the region. In addition, this research relies on self-reported survey responses, which are subject to recall bias and social desirability bias [26]. Furthermore, this study predominantly focuses on healthcare providers in one specific region, which may limit the generalizability of the results to other healthcare settings or regions with different healthcare infrastructure and resources.

## Conclusions

This study highlights the differences in understanding and practices between doctors and nurses, emphasizing the need for targeted educational interventions. Despite the identified gaps, a considerable proportion of healthcare providers demonstrate a good understanding of TIA definition and management. However, the lack of significant predictors for good knowledge and attitude suggests the need for more comprehensive strategies to enhance TIA management expertise across healthcare professionals. Further research with larger and more diverse samples, encompassing a wider geographic area, is essential to better comprehend the factors influencing TIA management and design effective interventions to bridge the existing gaps in care.
